# Mr. Denmark, in Answer to Mr. Chalmers

**Published:** 1807-07

**Authors:** Alex. Denmark

**Affiliations:** H. M. S. Qnebec


### *To the Editors of the Medical and Phyſical Journal.*

GENTLEMEN,

MY Paper on Gastritis, which you have done me the honour of inserting, I am sorry to find has involved the whole body of Naval Practitioners, by the *ungenerous* imputation of Mr. W. Chalmers, in a state of ignorance; and imputed to them a brutal apathy in the execution of their duties. Such aspersions cannot fail to awaken the suspicion of the *candid* and *unbiased* part of the profession; and to them will doubtless appear, as malevolent as well as unfounded.

On the present occasion, Gentlemen, I feel myself interested in a two-fold way:—In the first, as a member of that collective body which has been *declaimed* against; and in the next, as the individual who has been made the cause of such aspersions. Under colour of remarks upon my case, Mr. Chalmers has insidiously dared to calumniate the whole body of "Naval Practitioners." Had he confined his remarks to me alone, and criticized in a scientific way upon the case in question, he would have exhibited a genuine liberality of conduct, which will now require more than *his* ingenuity either to re-establish or retrieve. Could he not have confined himself to the discussion of a point which, to his mind, appeared so easily refutable, without descending to the most odious public invectives? It is saying little of such malicious traduction, to say simply, it is reprehensible. It goes to villify a class of men, whose merits, I hope, entitle them to more charitableness than your Correspondent seems blessed with. Unfortunately for him, his premises are false, and his conclusion not only becomes inert, but retorts upon himself. After such unmerited and pointed invectives, it cannot evince a want of decorum on my part, to affirm, he has been guilty of a breach of veracity. The purpose is known to himself; it is for me to make it known to others.

In replying to such a shameless libel, one requires to be armed with more than a common stock of stoicism, especially as we are in possession of the circumstances that instigated him to the use of such opprobrious acrimony. We discover a spirit of revenge against a service, in which the writer has been so lately, and so signally disgraced, and from which he suffered consequent expulsion. These are truths, Gentlemen, which, in justice to my my naval brethren, should be publicly promulgated, in order that, under such circumstances, your numerous and respectable readers may be enabled to decide, as to *his* being possessed of an "illiberal aversion to that class of men", (Naval Practitioners) whom he so very modestly introduces in his exordium. Now, judge whether revenge has any share in his criticism. However, I am of opinion, without such preliminary knowledge, Mr. Chalmers has unequivocally demonstrated to the world, by the unqualified diffuseness of his remarks, that he was not impelled either by purity of intention, or conscious veracity; motives, which on all occasions should be the entire guide of those who either write, commendably, for the information of others; or vainly, for the acquisition of fame.

The length of our late cruize has necessarily prevented my knowledge of Mr. W. Chalmers's attack upon my cases; and I do now confess, that a man who can be urged to criticize, by any other incentive than that of a wish to promote and disseminate knowledge, is, on his own account, unworthy of a reply. Nor would I deign to honour him so far, but that, on the ground of attacking an almost insignificant production of mine, he has most *unwarrantably* implicated a class of men, whose merits it would be unbecoming in me to neglect; but who have been honoured with unfeigned approbation from men, I presume, better qualified to judge of their deserts than Mr. Chalmers.

Here I have, in an individual capacity, to offer my most lively and unreserved acknowledgments to Dr. Boys for his eulogium, emanating from a benevolence of heart truly worthy of himself.

Mr. Chalmers commences with *flatly* saying, "My observations on the case want amendment. My treatment of the patient he conceived to be improper;" that, "the symptoms related by me were *certainly* those which indicate *pneumonia*;" and, "in delivering those symptoms, I have been inaccurate." To all these positive asseverations, I plead *not guilty*. And I humbly claim, Gentlemen, the patient and disspassionate attention of you, and the well-informed part of your multifarious readers, as an impartial tribunal, to hear and determine whether I cannot with facility, as *flatly* (but I trust in a more decorous manner) give the refutation to all he has said.

In the first place, in order to establish the correctness of my *diagnosis*, I would ask, whether the *pathognomic* symptoms of *pneumonia* are, "*Vomiting, very small and frequent*

*frequent pulse ; great prostration of strength ; pain on pressing the epigastric region ;* burning heat at the precordia ;"* and these symptoms becoming more and more aggravated. If they are, I give up my suit, and yield the palm to Mr. C.

Now, as to my inaccuracy " in delivering the symptoms," which belief seems to be occasioned by the pulse being 140 in frequency, on the first day, without any *increase* of heat on the surface : It would appear, Mr. Chalmers is not very conversant with the phenomena attendant on the cold fit of fever, and especially, in *sensitive* fever in gastritis, or he would express no incredulity on hearing the frequency of the patient's pulse to be 140, while at the same time the skin was natural, or, *colder than natural*, during that *stadium* of the disease. He is not aware that *irritative* fever is the precursor of *sensitive* ; and that the characteristic symptom of the former is *debility*, and that *debility* constitutes the *cold fit.* If you will give me leave, Gentlemen, I will explain more fully to the conception of Mr. C.

The long quiescence of the cutaneous vessels, occasioned by the subduction of their natural stimulus of heat, by the *continued* application of a *wet shirt* to the surface, in a suddenly reduced atmospheric temperature, would very satisfactorily account to the conviction of any one but Mr. C. for the frequency of pulse, and anhelation ; whilst, at the same time, the surface exhibited the *seemingly astonishing fact* of preserving itself *cool. Direct debility* was the consequence, induced by *irritative association* of the heart and pulmonary capillaries with those of the skin. The minute bronchial vessels being thus linked in action with the other parts of the aortal and the cutaneous capillaries, the circulation through them became impeded ; and the heart labouring to propel the blood through those collapsed vessels ineffectually, never perfectly emptied itself ; the diastole sooner recurred, and the pulsations became more frequent. In like manner was the anhelation produced by torpor, or quiescence of the pulmonary capillaries, from sympathy ; which Mr. C. confounds with the dyspnœa consequent on pulmonic inflammation, and occasioned by a *converse* state of those vessels, (plethora). He also confounds *frequency* with *velocity* of pulse, making them *synonimous ;* which on his part is incorrect. I infer

---

* In my Case it was printed *Hypogastric,* which error I beg leave to correct

I infer that the smallness and feebleness of the pulse• peculiarly inseparable from the sensitive fever in *Gastritis,* may be produced by the more direct sympathy which exists between the stomach and heart, than any other of the viscera.

A deficiency of all the secretions is consequent on the torpor already mentioned : hence the coldness of the skin, which so much excites your correspondent's disbelief: because, animal heat is produced, or evolved, in exact proportion to the existing quantity of secretions, and corresponding organism of the glands and capillaries.

The stupor and coma which are concomitants of pneumonia, may also be considered concomitants of Gastritis, and induced by the same means, namely, great expenditure of *sensorial power* on the inflamed organ; and perhaps, at the same time, aided by a deficient secretion of it.

He very candidly says, "he does not deny the *possibility* of the immense frequency of the pulse on the 1st day, nor its amounting to 160 on the 2d." Yet, "to him the statement seems incorrect." What downright sophistry!

My preconception ("as I call it") of the disease being Gastritis, was founded on the very great irritability of the stomach on the first morning of the disease, which *imme diately* rejected a small dose of antim. tart. This symptom *alone,* by the bye, would not have established that preconception, as it is a frequent concomitant of the cold stage of fevers: but viewed with those which occurred on the following day, (5th.) "*Vomiting and continued nausea, loathing both food and drink, greater prostration of strength, pulse increased to* 160, *and pain on pressing the epigastrium.* Mr. W. Chalmers himself, if he could for a moment divest himself of that prejudice by which he is so powerfully influenced; if he could enfranchise his ideas, and look with an unbiassed eye, *he, even he,* would be led to suspect the existence of Gastritis. And I doubt much, whether he has ever yet, backed by all his hyperbole, discovered these aggregate symptoms in real pneumonia. They are truly characteristic of Gastritis, and, together with having before seen the disease produced by the same means, viz. long application of cold to the surface, were surely such as to justify the *diagnosis.* Out of some hundred of pneumonic cases, of which I have had the treatment, I have never seen one where cough to a greater or less extent was not present.—Usher never coughed *once* during the whole of his illness.

Although open to conviction, it is not the mere ipse dixet et of Mr. Chalmers that can either convict me of "want of circumspection in the *diagnosis*, or rigor in the treatment;" not of "pneumonia," as he would have it, but of Gastritis. It is a happy circumstance that he is only a self-created arbiter, or, I fear his dispensations of justice could only be paralleled by his rooted antipathy "to that class of men," Naval Practitioners. I am reluctantly reduced to the necessity of this exposition. When unprovoked rancour exhausts all its means in exertions to fix a *general* stigma, forbearance becomes a dereliction of duty; and I hope never to give cause for an accusation of that magnitude, either in my public or private functions.

Now then, as to the treatment. Let me ask Mr. W. Chalmers (as he thinks proper to accuse me of "culpable timidity") in the name of humanity, whether he would have bled his patient to a greater extent than I did on the evening of the 1st day, after the supervention of "*spasmodic twitchings*, and approaching *syncope*?" Even admitting, for the sake of illustration, that the case was pneumonia, is it congruous to, or compatible with, the present improved state of medical science, to *produce syncope* by excess of *phlebotomy*? I believe not. If Mr. Chalmers would have done so, I do not wonder at his accusing me of timidity; but I think I should be void of feeling, were I not to commiserate the victim of such treatment.

I appeal to the *experienced* part of the medical world, whether I was right in administering an emetic on the first morning, when there was no sign by which I could prognosticate the existence of topical inflammation; and when the disease was taken for *simple fever*. Does Mr. C. know that an emetic, by inverting the action of the stomach, procures an accumulation of *sensorial power* in that viscus, (which may well be called the centre of sympathy with the rest of the body) and consequently gives subsequent vigour to the whole system, especially the heart and arteries, whose actions are particularly catenated with it? And does he also know, how much that effect contributes to the termination of the cold fit, and restoration of healthy action, and, by consequence, to the termination of the fever? If *he* is a stranger to these postulates, which are now generally received as facts, I am perfectly easy as to whether the "operation of the emetic appeared to *him* in a favourable light," or otherwise.

I conceive the exhibition of aromatics in gastritis, to be as inadmissable as opium, or alcohol; but my *liberal* readers will perceive, that it was on the evening of the fourth day of the disease, when "*pain and vomiting had subsided,*" when the "*pulse was smaller and feet cold,*" that Confect. arom. gr. v. opii. ʒß. was administered. And, the tottering which my ingenious commentator speaks of (when my patient attempted to walk) by wisely asking, was that to be wondered at? Why, I did not mention it as a wonderful phenomenon, but merely narrated it as a concomitant symptom of the disease, indicating *great debility*. He also enquires with pertinent shrewdness, "for what purpose he was suffered to *try* to walk?" It certainly could not be for the purpose of finding out whether he *could* walk or not; but as he did not go to bed on the first day, (our conveniences in a single decked ship not being such as to admit a convenient sick-birth in a cold climate) I happened to see him walk, and the symptom I conceived striking.

Have the appearances on dissection no weight with the incredulous and invidious Mr. C.? It was done in the presence of my assistant, and officers of the ship. In short, he has purposely perverted the whole case from beginning to end, and distorted it in such a manner, as not to be prototyped except by his own mind; if we may judge by the specimen he has just exhibited.

He is good enough to allow me the credit of not wishing to kill the man, by an equivocal implication.—He says, "All I did was *perhaps* well intended."—What a display of generosity!

He finds my corollaries (as he terms them) too abstruse for his penetration to expound. He evades it by referring me to his paper on Pneumonia. There, I fear, I shall look long in vain for any thing satisfactory. True, it is a production replete with words; but, inane, very inane as to matter. It is a long and laboured essay.—A pathological disquisition, displaying in the author a greater itch for writing than ability of execution. And his *Methodus Medendi,* it is admirable. Phlebotomy! What an important discovery!

That real pneumonia, Gentlemen, can ever be mistaken for typhus by the most unlettered *tyro* in the profession, I believe impossible; but, that typhus *may be,* and frequently *is,* taken for pneumonia, I have no doubt.

In truth, it appears our champion's chief talent is that of defamation, combined with a portion of temerity seldom to be met with; and utterly incompatible with the dignity and honourable character of the medical practitioner. Now, as he has so prostituted these essential requisites, and and stripped the profession of its richest cloathing, he must not be surprised if he discovers that he has drawn upon himself the just contempt of its votaries. And I shall say to such antagonists,

"Henceforth thou shalt digest the venom
"Of thy Spleen, tho' it do split thee."

Edentulus vescentium dentibus invidet.

I am, &c.

ALEX. DENMARK

*H. M. S. Quebec, March, 24,* 1807.

